# MIMU/Odometer Fusion with State Constraints for Vehicle Positioning during BeiDou Signal Outage: Testing and Results

**DOI:** 10.3390/s20082302

**Published:** 2020-04-17

**Authors:** Kai Zhu, Xuan Guo, Changhui Jiang, Yujingyang Xue, Yuanjun Li, Lin Han, Yuwei Chen

**Affiliations:** 1 School of Automobile and Traffic Engineering, Jiangsu University of Technology, Changzhou 213001, China; fatkyo@jsut.edu.cn (K.Z.); gx1836226090@163.com (X.G.); 2019500013@jsut.edu.cn (Y.X.); 2019560099@jsut.edu.cn (Y.L.); 2 Zhenjiang Zhongao AI Institute, Zhenjiang 212001, China; 3 School of Automation, Nanjing University of Science and Technology, Nanjing 210094, China; jiajialage@163.com; 4 Department of Photogrammetry and Remote Sensing, Finnish Geospatial Research Institute, Masala, FI-0245 Espoo, Finland

**Keywords:** GNSS, MIMU, odometer, state constraints

## Abstract

With the rapid development of autonomous vehicles, the demand for reliable positioning results is urgent. Currently, the ground vehicles heavily depend on the Global Navigation Satellite System (GNSS) and the Inertial Navigation System (INS) providing reliable and continuous navigation solutions. In dense urban areas, especially narrow streets with tall buildings, the GNSS signals are possibly blocked by the surrounding tall buildings, and under this condition, the geometry distribution of the in-view satellites is very poor, and the None-Line-Of-Sight (NLOS) and Multipath (MP) heavily affects the positioning accuracy. Further, the INS positioning errors will quickly diverge over time without the GNSS correction. Aiming at improving the position accuracy under signal challenging environment, in this paper, we developed an MIMU(Micro Inertial Measurement Unit)/Odometer integration system with vehicle state constraints (MO-C) for improving the vehicle positioning accuracy without GNSS. MIMU/Odometer integration model and the constrained measurements are given in detail. Several field tests were carried out for evaluating and assessing the MO-C system. The experiments were divided into two parts, firstly, field testing with data post-processing and real-time processing was carried out for fully assessing the performance of the MO-C system. Secondly, the MO-C was implemented in the BeiDou Satellite Navigation System (BDS)/integrated navigation system (INS) for evaluating the MO-C performance during the BDS signal outage. The MIMU standalone positioning accuracy was compared with that from the MIMU/Odometer integration (MO), MO-C and MIMU with constraints (M-C) for assessing the Odometer, and the influence of the constraint on the positioning errors reduction. The results showed that the latitude and longitude errors could be suppressed with Odometer assisting, and the height errors were suppressed while the state constraints were included.

## 1. Introduction

Unmanned Ground Vehicles (UGV) with complete automatic operation are regarded as the most promising technology to be available in the near future [1,2]. Precise and reliable position and navigation information are fundamental for the autonomous driving vehicles [3]. Currently, the Global Navigation Satellite System (GNSS) and the Inertial Navigation System (INS) are the most popular solutions for providing comparatively reliable positioning information [4]. GNSS is usually integrated with INS since they are highly complementary. The GNSS works by relying on the geometry distribution of the in-view satellites and signal quality, however, if the satellite signals are blocked by the surrendered buildings or obstacles, the GNSS will fail to generate precise positioning results [5]. Under this condition, the INS could provide moderate navigation solutions in a short time. However, due to the complex noises contained in the raw measurements from the gyroscope and accelerometer, the INS errors will accumulate quickly over time [4,5,6].

In the past decade, there has been a lot of literature focusing on improving the positioning accuracy under GNSS signal-challenging environments. These methods could be divided into two categories. The first solution is to suppress the INS noise and compensate for its positioning errors. The INS generates navigation solutions through processing the raw accelerator and gyroscope outputs. Limited by the manufacturing technology, there are complex noises contained in the raw measurements. Sheimy employed the Allan Variance method to characterize and quantify the noise [7,8]. Grip proposed an exponentially stable attitude and gyroscope bias estimation method in GNSS/INS integration [9]. Machine learning (LS-SVM, LSTM-RNN) methods were employed for modeling the errors [10,11,12,13]. Some calibration methods were also proposed to improve positioning accuracy [14,15,16,17,18]. Wu investigated the self-calibration of the Inertial Measurement Unit (IMU)/odometer integrated system for land vehicle navigation [14]. In addition, in the GNSS/INS integrated navigation system, some machine learning methods were employed and investigated to compensate for the INS errors during the GNSS signal outage [15,16,17,18]. These machine learning methods were well trained while the GNSS signal was normal.

The second solution is to employ more sensors in the GNSS/INS integration system and construct a multi-sensor fusion system. Among these sensors, LiDAR, vision cameras, altitude barometers, Chip Scale Atomic Clock (CSAC), and the odometer are the most popular sensors [19,20,21,22,23,24]. LiDAR is a sensor collecting the point cloud of the surrounding environment. With the continuous matching of the point cloud sequences, LiDAR can generate relative displacements and attitudes [19,20,21,22,23,24,25]. In aspects of the vision sensors, with the matching of the image’s sequences, attitude changes could also be extracted. With two well-calibrated vision cameras or depth cameras, this method could also provide positioning information [23,24]. An altitude barometer and odometer could provide height and odometer information, respectively. GNSS/LiDAR/HD-Map/INS integration system is a popular solution for autonomous driving vehicle positioning and navigation [25]. In addition, with the size and accuracy improvement, the CSAC is employed for augmenting the GNSS accuracy by providing a more precise frequency base [21]. 

In general, ground vehicles are usually equipped with an odometer for measuring the traveled distance. Therefore, it is convenient to develop a GNSS/MEMS-INS/odometer fusion system. Some works have revealed and demonstrated its effectiveness in improving positioning accuracy [26,27,28,29]. Georgy investigated the stochastic drift model of a MEMS (Micro-Electro-Mechanical System) gyroscope in a gyroscope/odometer/GPS integrated navigation system [26]. A mixture of particle filter and fuzzy neural network was employed for enhancing the MEMS-IMU/odometer/GPS integration for land vehicle applications [27,28]. An odometer and MEMS IMU were also employed for enhancing Precise Point Positioning (PPP) under weak satellite observability environments [29]. However, these studies were conducted while the GNSS was available, and the influence of the constraints and odometer on the positioning errors were not presented respectively and clearly. 

Scientists have explored and investigated this issue using the vehicle trajectory constraints to reduce the INS errors while the GNSS is unavailable [30,31,32,33]. Non-Holonomic Constraints (NHC) were employed as the measurements for suppressing the INS errors while GNSS was unavailable. While the vehicle is driving on the road, the velocity of both the up direction and perpendicular to the direction of vehicle traveling are almost zero [33]. The observability of the NHC was analyzed for demonstrating its effectiveness in land vehicle navigation systems [33]. However, NHC could not suppress the positioning errors in the forward direction. Therefore, in this paper, apart from the NHC, an odometer was also employed to suppress the positioning errors in the forward direction. We developed an MEMS-IMU/odometer integration navigation system considering the vehicle state constraints (MO-C) for ground vehicle positioning without GNSS. Both data post-processing and real-time processing experiments were carried out for assessing the navigation solution accuracy. Comparisons between MO-C, MO, and M-C were presented for evaluating and validating the odometer and constraints influence on the navigation solution accuracy. The contribution and innovation of this paper are summarized as follows:

(1) The model of the IMU/odometer with constraints is comprehensively given, detailed equations are listed and analyzed, and the influence of the odometer and constraints on the positioning errors were numerically compared and evaluated, which might be a reference for implementing these algorithms for different conditions.
(2) The odometer and constraints were firstly implemented in a BeiDou Satellite Navigation System (BDS)/MIMU loosely-integrated navigation system for evaluating its performance and effectiveness in reducing and suppressing INS positioning errors while GNSS was unavailable, and positioning errors were presented for assessing these methods’ feasibility in a GNSS/INS integration framework.
(3) In the experiments, both post-processing and real-time filed tests were carried out for assessing the odometer and NHC performance in improving the positioning accuracy, respectively, and the NHC and odometer were employed in the BDS/MIMU integrated navigation system, which was of great significance for improving the positioning accuracy during the BDS signal outage.

The rest of the paper is organized as follows: Section 2 introduces the model of the MO, MO-C (state measurement equations), the integration filter, and the vehicle state detection method. Section 3 presents the results and numerical analysis of the field tests. Then, we discuss and conclude the paper, and the limitations and the future work are detailed.

## 2. Model 

### 2.1. GNSS/MIMU Loose Integration Model 

The state vector of the GNSS/MIMU loose integration model contains 15 states, and the state vector $X_{I}$ is defined as:

(1) $$X_{I}={\left[\delta \phi ,\delta v,\delta p,\delta \varepsilon ,\delta \nabla \right]}^{T}$$

where δϕ=[α,β,γ] denotes the three-axis attitude errors (pitch, roll, and yaw angle errors), $\delta v=\left[\delta v_{e},\delta v_{n},\delta v_{u}\right]$ denotes the three-axis velocity errors (east, north, and up velocity errors) in the ENU navigation frames, δp=[δL,δλ,δh] denotes the three-axis positioning errors (latitude, longitude, and height errors), $\delta \varepsilon =\left[\varepsilon_{x},\varepsilon_{y},\varepsilon_{z}\right]$ denotes the bias errors of the three-axis gyroscopes in body frame, and $\delta \nabla =\left[\nabla_{x},\nabla_{y},\nabla_{z}\right]$ denotes the bias errors of the three-axis accelerometers.

The state equation GNSS/MIMU loose integration can be written as:

(2) $${\dot{X}}_{I}\left(t\right)=F_{I}\left(t\right)\cdot X_{I}\left(t\right)+G_{I}\left(t\right)W_{I}\left(t\right)$$

where $F_{I}\left(t\right)$ is the state transferring matrix; $W_{I}$ denotes the state model noise matrix [18,19,20,21]. Specifically, the detailed description of the state equation is as:

(3) $$\left[\begin{array}{l} \delta {\dot{p}}_{3\times 1}\\ \delta {\dot{v}}_{3\times 1}\\ \delta {\dot{\phi }}_{3\times 1}\\ {\dot{\varepsilon }}_{3\times 1}\\ {\dot{\nabla }}_{3\times 1} \end{array}\right]=\left[\begin{array}{lllll} F_{pp} & F_{pv} & 0_{3\times 3} & 0_{3\times 3} & 0_{3\times 3}\\ F_{vp} & F_{vv} & F_{v\phi } & 0_{3\times 3} & C_{b}^{n}\\ 0_{3\times 3} & 0_{3\times 3} & F_{\phi \phi } & C_{b}^{n} & 0_{3\times 3}\\ 0_{3\times 3} & 0_{3\times 3} & 0_{3\times 3} & F_{\varepsilon \varepsilon } & 0_{3\times 3}\\ 0_{3\times 3} & 0_{3\times 3} & 0_{3\times 3} & 0_{3\times 3} & F_{\nabla \nabla } \end{array}\right]\left[\begin{array}{l} \delta p_{3\times 1}\\ \delta v_{3\times 1}\\ \delta \phi_{3\times 1}\\ \varepsilon_{3\times 1}\\ \nabla_{3\times 1} \end{array}\right]+\left[\begin{array}{llll} 0_{3\times 3} & 0_{3\times 3} & 0_{3\times 3} & 0_{3\times 3}\\ C_{b}^{n} & 0_{3\times 3} & 0_{3\times 3} & 0_{3\times 3}\\ 0_{3\times 3} & C_{b}^{n} & 0_{3\times 3} & 0_{3\times 3}\\ 0_{3\times 3} & 0_{3\times 3} & I_{3\times 3} & 0_{3\times 3}\\ 0_{3\times 3} & 0_{3\times 3} & 0_{3\times 3} & I_{3\times 3} \end{array}\right]\left[\begin{array}{l} w_{3\times 1}^{v}\\ w_{3\times 1}^{\phi }\\ w_{3\times 1}^{\varepsilon }\\ w_{3\times 1}^{\nabla } \end{array}\right]$$

Further, the first-order discrete form of the state equation is as:

(4) $${\dot{X}}_{I}\left(k+1\right)=\left(I+F_{I}\cdot T\right)\cdot X_{I}\left(k+1\right)+G_{I}\cdot T\cdot W_{I}\left(k+1\right)$$

(5) $$\begin{array}{l} \left[\begin{array}{l} \delta {\dot{p}}_{3\times 1}\\ \delta {\dot{v}}_{3\times 1}\\ \delta {\dot{\phi }}_{3\times 1}\\ {\dot{\varepsilon }}_{3\times 1}\\ {\dot{\nabla }}_{3\times 1} \end{array}\right]=\left[\begin{array}{ccccc} I_{3\times 3}+F_{pp} & F_{pv}T & 0_{3\times 3} & 0_{3\times 3} & 0_{3\times 3}\\ F_{vp}T & I_{3\times 3}+F_{vv}T & F_{v\phi }T & 0_{3\times 3} & C_{b}^{n}T\\ 0_{3\times 3} & 0_{3\times 3} & I_{3\times 3}+F_{\phi \phi }T & C_{b}^{n}T & 0_{3\times 3}\\ 0_{3\times 3} & 0_{3\times 3} & 0_{3\times 3} & I_{3\times 3}+F_{\varepsilon \varepsilon }T & 0_{3\times 3}\\ 0_{3\times 3} & 0_{3\times 3} & 0_{3\times 3} & 0_{3\times 3} & I_{3\times 3}+F_{\nabla \nabla }T \end{array}\right]\left[\begin{array}{l} \delta p_{3\times 1}\\ \delta v_{3\times 1}\\ \delta \phi_{3\times 1}\\ \varepsilon_{3\times 1}\\ \nabla_{3\times 1} \end{array}\right]\\ +\left[\begin{array}{llll} 0_{3\times 3} & 0_{3\times 3} & 0_{3\times 3} & 0_{3\times 3}\\ C_{b}^{n}T & 0_{3\times 3} & 0_{3\times 3} & 0_{3\times 3}\\ 0_{3\times 3} & C_{b}^{n}T & 0_{3\times 3} & 0_{3\times 3}\\ 0_{3\times 3} & 0_{3\times 3} & I_{3\times 3}T & 0_{3\times 3}\\ 0_{3\times 3} & 0_{3\times 3} & 0_{3\times 3} & I_{3\times 3}T \end{array}\right]\left[\begin{array}{l} w_{3\times 1}^{v}\\ w_{3\times 1}^{\phi }\\ w_{3\times 1}^{\varepsilon }\\ w_{3\times 1}^{\nabla } \end{array}\right] \end{array}$$

where T denotes the integration filter updating interval. 

Then, the measurement model of the GNSS/MIMU integration is expressed as:

(6) $$Z_{k+1}=H_{k+1}X_{k+1}+\mu_{k+1}$$

where $Z_{k+1}$ denotes the measurement matrix, $H_{k+1}$ denotes the observation matrix, and $\mu_{k+1}$ denotes the measurement noise. In the loose integration model, the measurement vector is composed of GNSS and MIMU position and velocity difference, and the detailed description is as:

(7) $$Z_{k+1}=\left[\begin{array}{c} \left(L_{k+1}^{MIMU}-L_{k+1}^{GNSS}\right)\cdot \left(R_{M}+h\right)\\ \left(\lambda_{k+1}^{MIMU}-\lambda_{k+1}^{GNSS}\right)\cdot \left(R_{N}+h\right)cos\left(L\right)\\ h_{k+1}^{MIMU}-h_{k+1}^{GNSS}\\ v_{e,k+1}^{MIMU}-v_{e,k+1}^{GNSS}\\ v_{n,k+1}^{MIMU}-v_{n,k+1}^{GNSS}\\ v_{u,k+1}^{MIMU}-v_{u,k+1}^{GNSS} \end{array}\right]$$

A detailed description of the observation matrix $H_{k+1}$ is:

(8) $$Z_{k+1}=\left[\begin{array}{cc} diag\left[R_{M}+h\left(R_{N}+h\right)\cos\left(L\right)\;1\right] & 0_{3\times 3}\;0_{3\times 3}\\ 0_{3\times 3} & 0_{3\times 3} \end{array}\right]X_{k+1}+\mu_{k+1}$$

### 2.2. State Constraints

**Figure 1:**
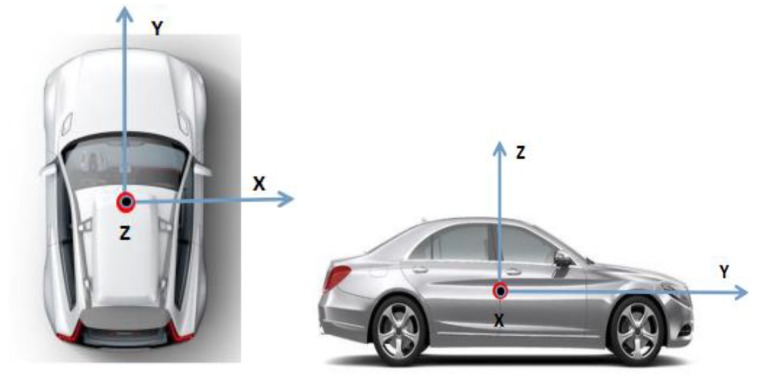
Illustration of the vehicle body coordinates.

The definition of the vehicle body coordinates is presented in Figure 1. In the coordinates, the origin is the center of gravity of the vehicle body, the Y axis points to the direction of the vehicle traveling, the X axis points to the right side of the vehicle body, and the Z axis points to the up direction of the vehicle.

According to the driving characteristics of the vehicle on the road, while the vehicle is running normally on the road without sideslip or jump, e.g., the vehicle is driving on an expressway, the X-axis and Z-axis speeds of the vehicle in the defined vehicle frame are approximately zero. The characteristic is modeled as:

(9) $$\{\begin{array}{l} V_{x}^{v}\approx 0\\ V_{z}^{v}\approx 0 \end{array}$$

In the dynamic trajectory, vehicle kinematic constraints are employed for suppressing the diverging positioning errors under the GNSS-denied environment. Vehicle kinematic constraints are constructed in the vehicle body coordinates. Although the Inertial Measurement Unit (IMU) is installed on the vehicle body, there is usually a misalignment angle between the IMU body coordinates and the vehicle body coordinates. The misalignment angles will affect the velocity constraints listed in Equation (1). 

Assuming the conversion matrix between the IMU body coordinates and the vehicle body system is $C_{v}^{b}$. The heading misalignment angle is $\alpha_{\psi }$, the pitch misalignment angle is $\alpha_{\theta }$, and the roll misalignment angle is $\alpha_{\gamma }$. Converting the velocity in the IMU body system to the vehicle body system can be modeled as:

(10) $$V^{b}=C_{v}^{b}V^{v}$$

where $V^{v}$ is the velocity vector in the IMU body coordinates, and $V^{b}$ is the velocity vector in vehicle body coordinates. 

Specifically,

(11) $$V^{b}=\left[\begin{array}{c} V_{x}^{b}\\ V_{y}^{b}\\ V_{z}^{b} \end{array}\right]=C_{v}^{b}\left[\begin{array}{c} V_{x}^{v}\\ V_{y}^{v}\\ V_{z}^{v} \end{array}\right]=\left[\begin{array}{c} \sin\alpha_{\psi }\cos\alpha_{\theta }\\ \cos\alpha_{\psi }\cos\alpha_{\theta }\\ \sin\alpha_{\theta } \end{array}\right]V_{y}^{v}$$

While the vehicle is moving, the $V_{y}^{v}$ is not zero. The $V_{y}^{v}$ will be projected to the $V_{x}^{b}$ and $V_{z}^{b}$ through the heading and pitch misalignment angle. The roll angle does not influence the $V_{x}^{b}$ and $V_{z}^{b}$. The influence of the misalignment angle on the velocity $V_{x}^{b}$ and $V_{z}^{b}$ can be described as

(12) $$\delta C_{b}^{v}=-\left[\begin{array}{c} \delta \alpha_{\theta }\\ 0\\ \delta \alpha_{\psi } \end{array}\right]\times C_{b}^{v}=-\delta \alpha \times C_{b}^{v}$$

While employing the constraints in the GNSS/MIMU loose integration, the misalignment angle between the MIMU body frame and the vehicle body frame should be considered and added to the state vector. The new state vector is as:

(13) $$X={\left[\begin{array}{cc} X_{I} & X_{\alpha } \end{array}\right]}^{T}$$

where $X_{I}$ is the same as that in Equation (1), $X_{\alpha }={\left[\delta \alpha_{\theta }\;\delta \alpha_{\psi }\right]}^{T}$, $\delta \alpha_{\theta }$ is the misalignment heading angle, and the $\delta \alpha_{\psi }$ is the misalignment pitch angle.

Once the IMU is fixed on the vehicle, the misalignment angles can be regarded as constant values. Therefore, the first-order derivative of the misalignment angles is zero.

(14) $$\{\begin{array}{l} \delta {\dot{\alpha }}_{\theta }=0\\ \delta {\dot{\alpha }}_{\psi }=0 \end{array}$$

Then, the state equation of the MIMU can be as:

(15) $$\begin{array}{ll} \dot{X}(t) & =\left[\begin{array}{c} {\dot{X}}_{I}(t)\\ {\dot{X}}_{\alpha }(t) \end{array}\right]=\left[\begin{array}{cc} F_{I}(t) & 0\\ 0 & F_{\alpha }(t) \end{array}\right]\left[\begin{array}{c} X_{I}(t)\\ X_{\alpha }(t) \end{array}\right]+\left[\begin{array}{cc} G_{I}(t) & 0\\ 0 & G_{\alpha }(t) \end{array}\right]\left[\begin{array}{c} W_{I}(t)\\ W_{\alpha }(t) \end{array}\right]\\ & =F(t)\cdot X(t)+G(t)\cdot W(t) \end{array}$$

where $F_{I}(t)$ is the state transformation matrix of the IMU’s states,$F_{\alpha }(t)$ is the state transformation matrix of the misalignment angles, $W_{I}(t)$ is the IMU state noise matrix, and $W_{\alpha }(t)$ is the misalignment angle state noise matrix. 

Commonly, the MIMU and GNSS loose integration model is constructed in East–North–Up (ENU) coordinates. Positioning and velocity information from the GNSS and INS are subtracted and employed as the measurement information. Converting the SINS velocity from the ENU coordinates to the vehicle body frame.

(16) $$V^{v}=C_{b}^{v}C_{n}^{b}V^{n}$$

where $C_{b}^{v}$ means the velocity conversion from the vehicle body frame from the IMU body coordinates, $C_{n}^{b}$ is the velocity transformation matrix from the ENU navigation frame to the vehicle body coordinates, and $V^{n}$ is the velocity vector in the ENU navigation frame.

Combining Equations (11)–(16), the differential equation is

(17) $$\begin{array}{ll} \delta V^{v} & =C_{b}^{v}\left(C_{n}^{b}\varphi \times V^{n}+C_{n}^{b}\delta V^{n}\right)-\delta \alpha \times C_{b}^{v}C_{n}^{b}V^{n}\\ & =\left(-C_{b}^{v}C_{n}^{b}\left(V^{n}\right)\times \right)\varphi +C_{b}^{v}C_{n}^{b}\delta V^{n}+\left(\left(C_{b}^{v}C_{n}^{b}V^{n}\right)\times \right)\delta \alpha \\ & =M_{3\times 3}^{1}\varphi +M_{3\times 3}^{2}\delta V^{n}+M_{3\times 3}^{3}\delta \alpha \end{array}$$

The measurement equation is

(18) $$Z_{V}=\left[\begin{array}{c} V_{x}^{v}-0\\ V_{z}^{v}-0 \end{array}\right]=H_{V}X+V_{V}$$

where $H_{V}$ is the measurement matrix, and the $V_{V}$ is the noise matrix.

(19) $$H_{V}=\left[\begin{array}{ccccc} M_{3\times 3}^{1}\left(1,\times \right) & M_{3\times 3}^{2}\left(1,\times \right) & 0_{1\times 11} & 0 & M_{3\times 3}^{3}\left(1,3\right)\\ M_{3\times 3}^{1}\left(3,\times \right) & M_{3\times 3}^{2}\left(3,\times \right) & 0_{1\times 11} & M_{3\times 3}^{3}\left(3,1\right) & 0 \end{array}\right]$$

where $M_{3\times 3}^{1}\left(1,\times \right)$ is the first row of the matrix $M_{3\times 3}^{1}$, $M_{3\times 3}^{3}\left(1,3\right)$ is the element in the first row and third column, $M_{3\times 3}^{1}\left(3,\times \right)$ is the third row of the matrix $M_{3\times 3}^{1}$, and $M_{3\times 3}^{3}\left(3,1\right)$ is the element in the third row and first column.

### 2.3. MIMU/Odometer Measurement Model 

The state vector of the MIMU/Odometer integration model is the same as Equations (13)–(15), however, the measurement equation is different. The odometer output is listed as:

(20) $${\hat{V}}_{odo}^{b}={\left[\begin{array}{ccc} 0 & {\hat{V}}_{odoy}^{b} & 0 \end{array}\right]}^{T}$$

We then convert the odometer velocity from the IMU body frame to the ENU navigation frame, then subtracting them with the velocity from the MIMU. The equation is listed as:

(21) $$Z_{O}=V_{I}^{n}-C_{b}^{n}{\hat{V}}_{odo}^{b}=\left[\begin{array}{c} V_{IE}-V_{odoE}^{n}\\ V_{IN}-V_{odoN}^{n}\\ V_{IU}-V_{odoU}^{n} \end{array}\right]=H_{O}X+V_{O}$$

where $V_{I}^{n}$ is the velocity from the MIMU, and ${\hat{V}}_{odo}^{b}$ is the velocity from the odometer, $H_{O}$ is the measurement matrix, and $V_{O}$ is the noise matrix.

(22) $$H_{O}=\left[\begin{array}{ccccc} 0_{1\times 3} & 1 & 0 & 0 & 0_{1\times 11}\\ 0_{1\times 3} & 0 & 1 & 0 & 0_{1\times 11}\\ 0_{1\times 3} & 0 & 0 & 1 & 0_{1\times 11} \end{array}\right]$$

### 2.4. MIMU/Odometer Measurement Model with Constraints 

Combining Equations (8)–(14), the MIMU/odometer measurement model with constraints is as:

(23) $$Z_{OV}=\left[\begin{array}{c} V_{x}^{v}-0\\ V_{IN}-V_{odoN}^{n}\\ V_{z}^{v}-0 \end{array}\right]=H_{OV}X+V_{OV}$$

where $V_{x}^{v}$ is the X-axis velocity in the vehicle body coordinates, $V_{z}^{v}$ is the Z-axis velocity in the vehicle body coordinates, $H_{OV}$ is the measurement matrix, and $V_{OV}$ is the measurement noise matrix.

(24) $$H_{OV}=\left[\begin{array}{ccccccc} M_{3\times 3}^{1}\left(1,\times \right) & M_{3\times 3}^{2}\left(1,1\right) & M_{3\times 3}^{2}\left(1,2\right) & M_{3\times 3}^{2}\left(1,3\right) & 0_{1\times 11} & 0 & M_{3\times 3}^{3}\left(1,3\right)\\ 0_{1\times 3} & 0 & 1 & 0 & 0_{1\times 11} & 0 & 0\\ M_{3\times 3}^{1}\left(3,\times \right) & M_{3\times 3}^{2}\left(3,1\right) & M_{3\times 3}^{2}\left(3,2\right) & M_{3\times 3}^{2}\left(3,3\right) & 0_{1\times 11} & M_{3\times 3}^{3}\left(3,1\right) & 0 \end{array}\right]$$

### 2.5. Integration Method 

The state model is listed in Equations (5)–(7), and the measurement models under different conditions are given in Equations (10), (14), and (15). Here, a Kalman filter is employed for carrying out the integration. The Kalman filter is as: 

The Kalman filter state vector and state covariance prediction are as:

(25) $${\hat{X}}_{k}^{-}=\Phi_{k|k-1}{\hat{X}}_{k-1}$$

(26) $$P_{k}^{-}=\Phi_{k|k-1}P_{k-1}\Phi_{k|k-1}^{T}+Q_{k-1}$$

The updating of the gain matrix, state vector, and the covariance are as follows:

(27) $$K_{k}=P_{k}^{-}H_{k}^{T}{\left(H_{k}P_{k}^{-}H_{k}^{T}+R_{k}\right)}^{-1}$$

(28) $${\hat{X}}_{k}={\hat{X}}_{k}^{-}+K_{k}(Z_{k}-H_{k}{\hat{X}}_{k}^{-})$$

(29) $$P_{k}=\left(I-K_{k}H_{k}\right)P_{K}^{-}$$

where $\Phi_{k|k-1}$ is the state transformation matrix; ${\hat{X}}_{k}^{-}$ is the predicted state vector through the state transformation matrix and the state vector at previous epoch; $P_{k}^{-}$ is the covariance matrix; $K_{k}$ is the gain matrix at the $k_{th}$ epoch, which decides the updating weight between the predicted state vector and the new measurements; ${\hat{X}}_{k}$ is the estimated state vector at the $k_{th}$ epoch; $P_{k}$ is the covariance matrix.

**Figure 2:**
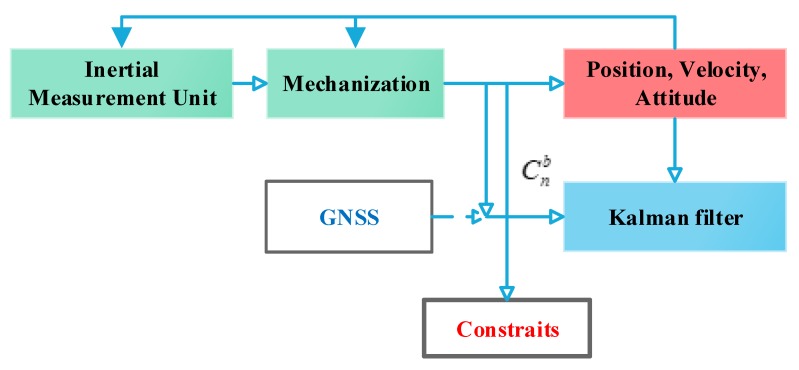
Structure of the Global Navigation Satellite System (GNSS)/Inertial Navigation System (INS)/odometer/Non-Holonomic Constraints (NHC) integrated system.

Based on the above model, Figure 2 shows the structure of the integration system with constraints. When the GNSS is available, the GNSS/MIMU integration system can provide satisfying navigation solutions. While GNSS is unavailable, constraints are employed in the integration system for estimating the IMU state errors and compensating them. 

## 3. Results

**Figure 3:**
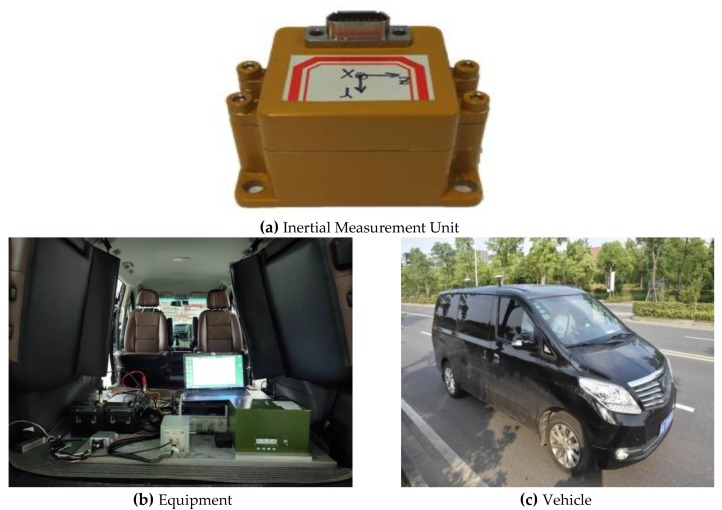
Field testing equipment.

For fully testing and assessing the performance of the MO-C system, two different field tests were carried out. The equipment employed in the field testing are given in Figure 3, the employed MIMU is presented in Figure 3a, and the vehicle is shown in Figure 3b,c. In the first field test, the MIMU and Odometer dataset was collected and post-processed through the software implemented in Matlab [34]. In the second field test, the algorithm was implemented using the hardware platform DSP+FPGA (Digital Signal Processor, DSP; Field Programmable Gate Array, FPGA), and the results were obtained from real-time processing of the data. The MO-C was also implemented in the BDS/MIMU integrated navigation system for improving the effectiveness during BDS signal outage. 

Parameters of the employed MIMU were given in Table 1. The gyroscope bias stability was 3 degrees/h, and the accelerometer bias stability was 0.1 mg. The MIMU sampling frequency was 400 Hz, and the odometer data output frequency was 20 Hz. The integration filter operation period was one second. 

### 3.1. Field Testing with Data Post-Processing

**Figure 4:**
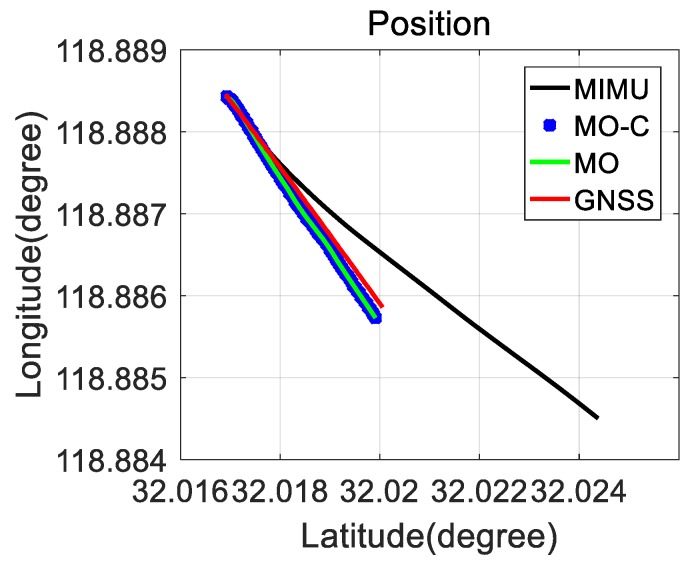
The trajectory from different methods.

**Figure 5:**
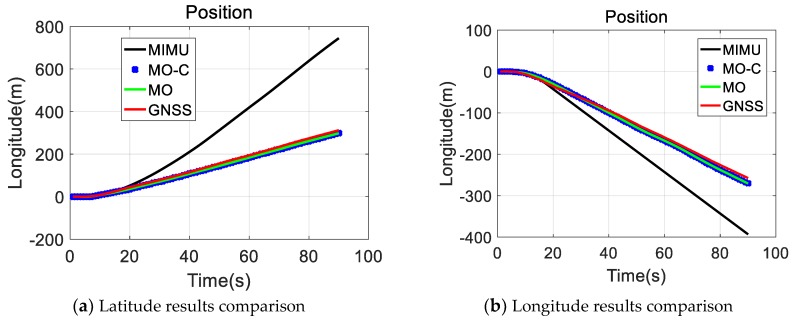

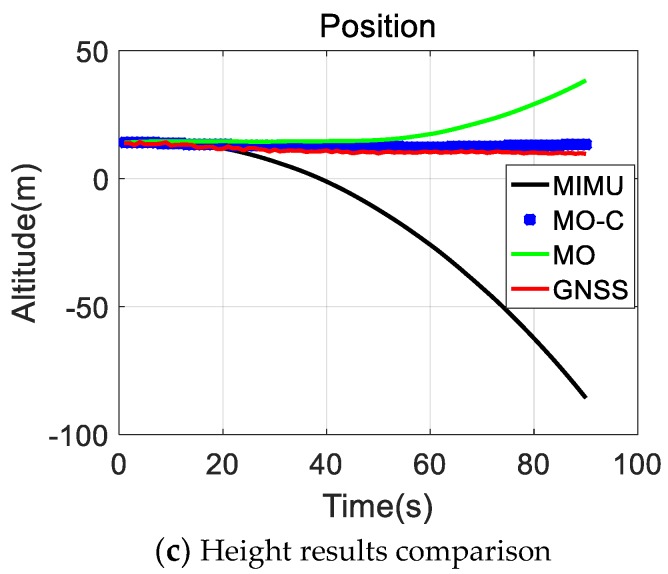
Positioning results comparison.

**Figure 6:**
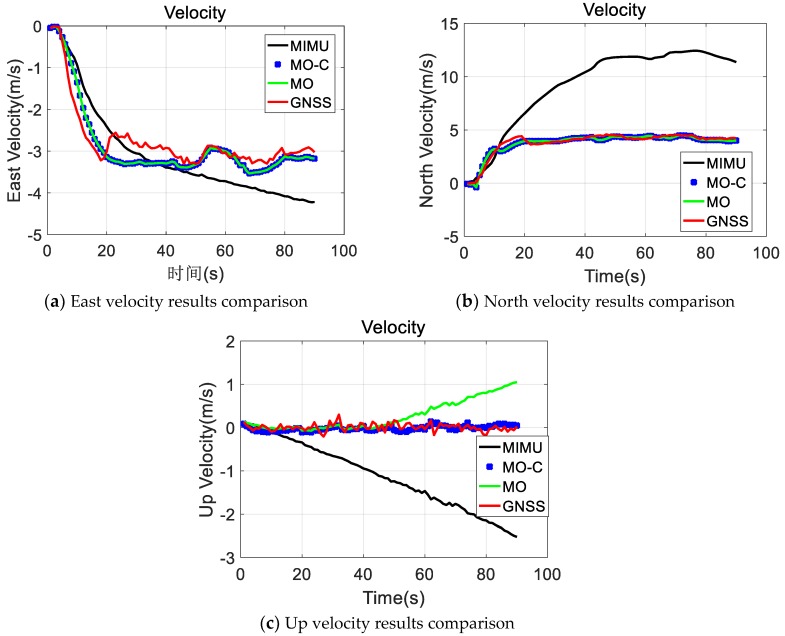
Velocity results comparison.

MIMU and Odometer data were collected with the equipment presented in Figure 3. Following positioning values comparisons ( GNSS, MIMU, MIMU/odometer (MO), and MIMU/Odometer with constraints (MO-C) are presented in Figure 4, Figure 5 and Figure 6. Trajectories obtained from different methods were presented in Figure 4. The MO-C and MO results were similar in this trajectory. The positioning curves in Figure 5 are plotted as the trajectory. Velocity results comparisons are presented in Figure 6. Positioning and velocity error analysis including the Root Mean Square Error (RMSE) and 90-s errors without GNSS are listed in Table 2. Through a comparison of the results of the GNSS, MIMU, MO, and MO-C, it could be seen that:

(1) Compared with the MIMU standalone, the positioning errors were suppressed with the odometer and constraints included, the latitude and longitude curves were almost consistent with the GNSS curves. The east and north velocity were also consistent with the GNSS results. The height and up velocity were also converging over time.
(2) Compared with the MIMU standalone, within 90 s, the MO and MO-C latitude errors reduced by 98.8% and 98.9%; the MO and MO-C longitude errors reduced by 95.1% and 95.5%; the MO and MO-C height errors decreased by 81.1% and 95.9%. In aspects of the velocity errors, both MO and MO-C east velocity errors reduced by 87.2%; the MO and MO-C north velocity errors decreased by 96.8% and 96.9%; the MO and MO-C up velocity obtained a 63.4% and 99.2% improvement. Among them, the up direction position and velocity errors obtained the largest reduction compared with that of other directions.

### 3.2. Field Testing with Real-Time Data Processing 

After the post-processing field testing, we carried out real-time data processing-based field testing for further evaluation of the performance of the method. The algorithm was implemented using the DSP+FPGA hardware platform with real-time processing data fusion. This sub-section is divided into four parts. In the first part, we evaluate the MIMU with constraints; in the second part, the MIMU/odometer integration is assessed; in the third part, the MO-C results are presented and analyzed; in the last part, the MO-C was integrated into GNSS/MIMU integrated navigation system, and the navigation solutions are presented and compared during a signal outage.

#### 3.2.1. MIMU with Constraints

**Figure 7:**
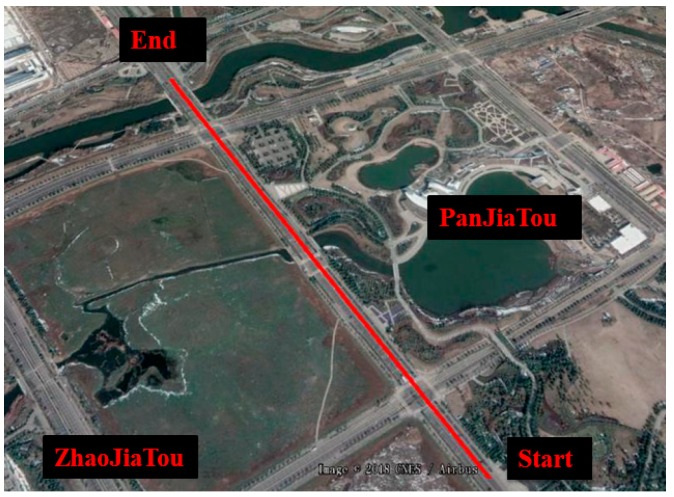
Field testing trajectory.

**Figure 8:**
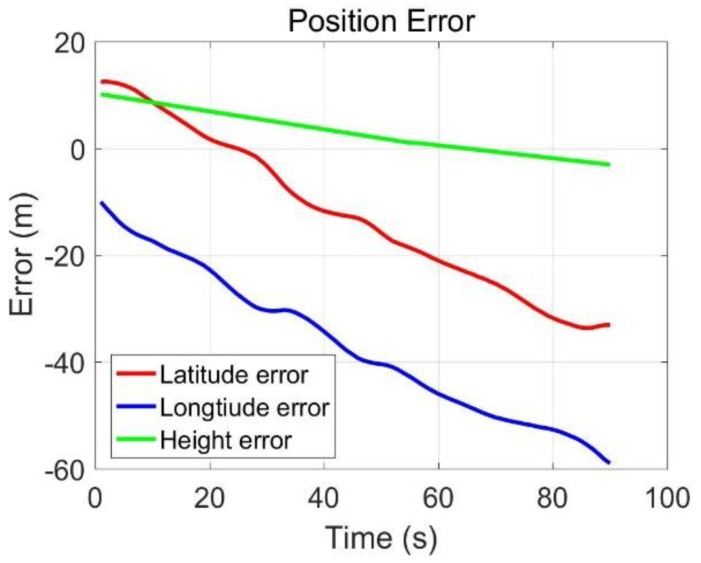
Three-axis positioning errors.

**Figure 9:**
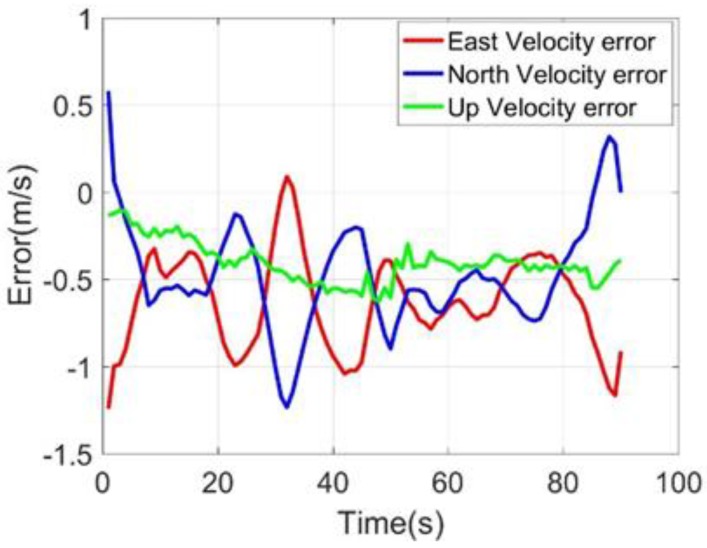
Three-axis velocity errors.

**Figure 10:**
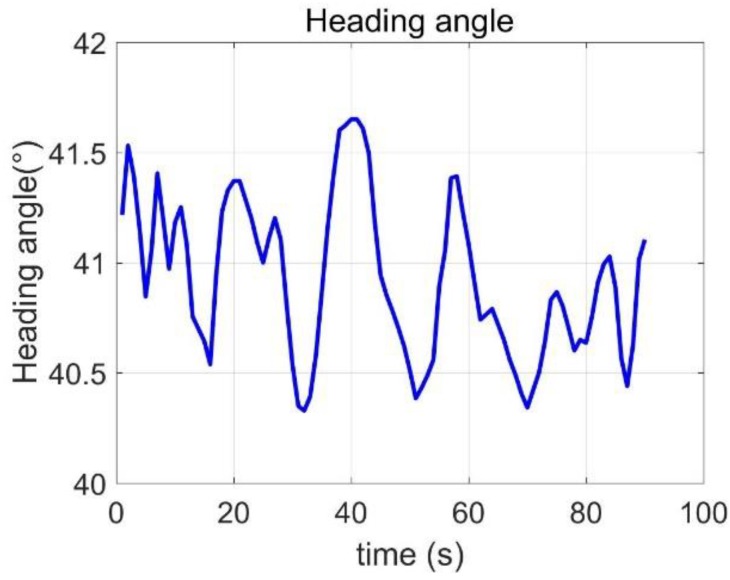
Heading angle.

The field-testing trajectory was presented in Figure 7, and the positioning errors and velocity errors were presented in Figure 8 and Figure 9. The latitude and longitude errors of M-C gradually accumulated, but gradually tended to be flat, the altitude errors gradually stabilized, and the errors were small; although the three-dimensional speed errors were stable, the east and north speed errors were relatively large, and the up speed errors were small. Without GNSS, the position and velocity errors within 90 s were as follows: latitude error was −25.85 m, longitude error was −28.80 m, altitude error was −3.06 m, east velocity error was −0.91 m/s, north velocity error was 0.27 m/s, up velocity error was −0.38 m/s. The results showed that the constraints were effective for suppressing the errors of the MIMU in the dynamic trajectory. However, the M-C 90-s error values were still not ideal, which was also affected by the heading angle. The heading angle was presented in Figure 10, and it varied between 40° and 42°. The vehicle kinematics constraints could only suppress the X-axis and Z-axis position and velocity errors. 

#### 3.2.2. MIMU/Odometer Integration 

**Figure 11:**
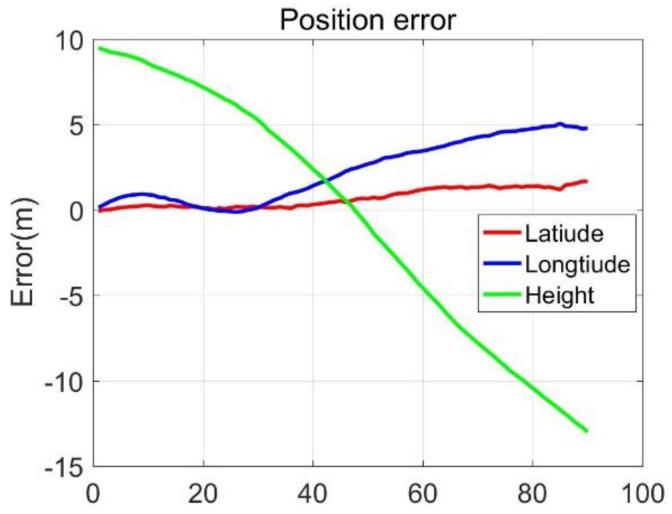
Positioning errors.

**Figure 12:**
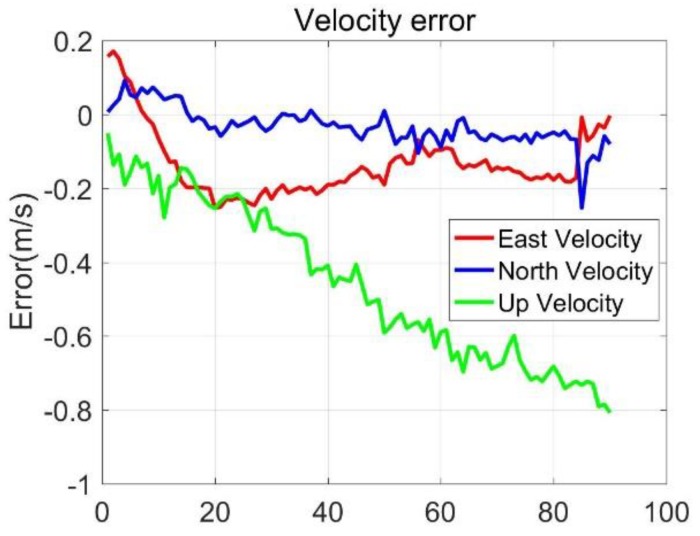
Velocity errors.

Three-axis position errors and velocity errors were presented in Figure 11 and Figure 12, and it could be seen that the MO latitude and longitude errors were stable and small, the altitude errors firstly decreased and then increased; the east and north speed errors were stable, and kept within 0.2 m/s, the up velocity errors gradually increased, and it trended to diverge. The 90-s position and velocity errors were as follows: latitude error was 1.74 m, longitude error was 4.73 m, altitude error was −13.35 m, east velocity error was 0.03 m/s, north velocity error was −0.06 m/s, and the up velocity error was −0.85 m/s. The results showed that the MO was effective for reducing the MIMU positioning errors. It was worth of noting that the error of up velocity increased gradually without good constraints, which was the same as the results in Section 3.1. If we wanted to obtain high-precision three-dimensional positioning and speed measurement under GNSS-denied environments, additional sensors or methods were necessary to suppress the height and up-direction velocity errors. 

#### 3.2.3. MIMU/Odometer Integration with Constraints 

**Figure 13:**
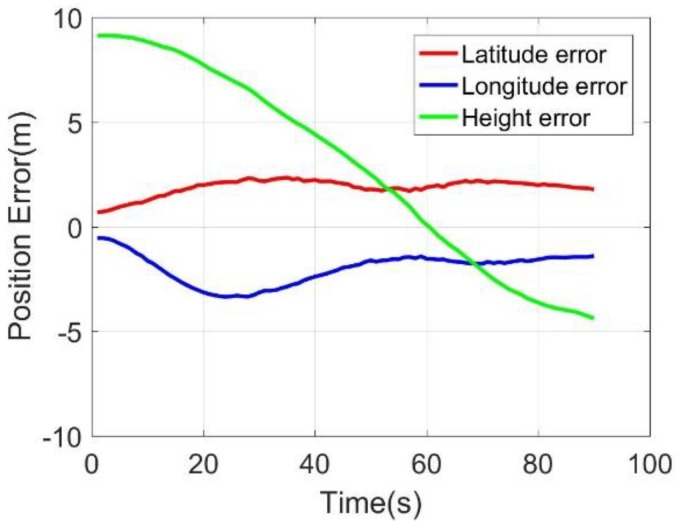
Positioning errors.

**Figure 14:**
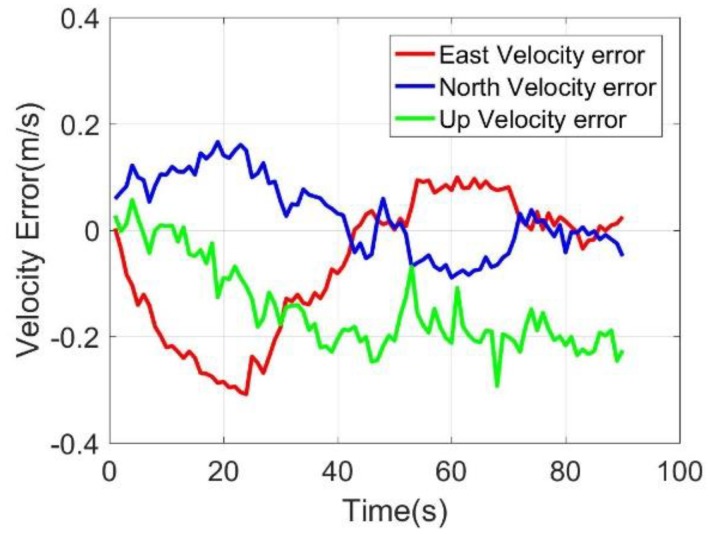
Velocity errors.

In Figure 13 and Figure 14, the three-dimensional position and velocity errors of MO-C were stable and small. The height errors and the up velocity errors were significantly smaller than that of the MC. The added kinematic constraints information performed well in suppressing the up velocity errors and height errors. The 90-s position and velocity errors were as follows: latitude error was 1.72 m, longitude error was −1.36 m, altitude error was −4.38 m; east velocity error was 0.02 m/s, north velocity error was −0.04 m/s, and up velocity error was −0.23 m/s. The position and velocity errors comparison for MINS/BDS, M-C, MO, and MO-C are listed in Table 3. It could be seen that the latitude and longitude errors were suppressed while the odometer was included in the system. With the odometer assisting, the MO-C 90-s latitude and longitude errors decreased over 90% compared with that of M-C. While adding the constraints to the MO, the MO-C longitude and height errors performed with a 71.2% and 67.2% decrease, and the north and up velocity decreased by 33.3% and 72.9%. These results demonstrated the effectiveness of the odometer and constraints in position and velocity error suppression. 

#### 3.2.4. Implementation of MC-O in MIMU/BDS during Signal Outage 

**Figure 15:**
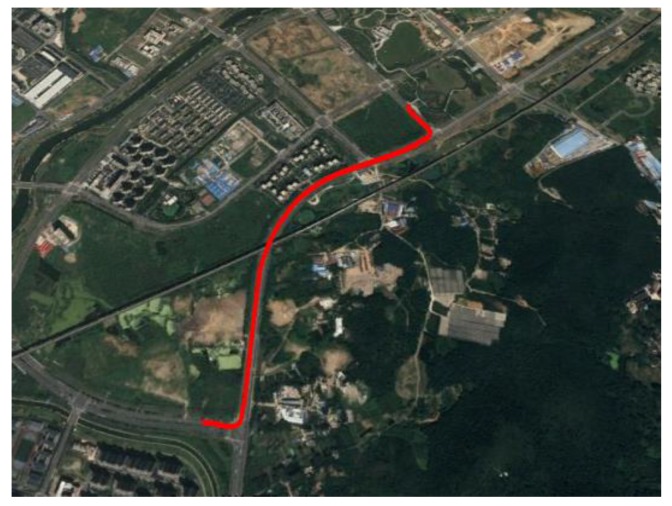
Trajectory.

**Figure 16:**
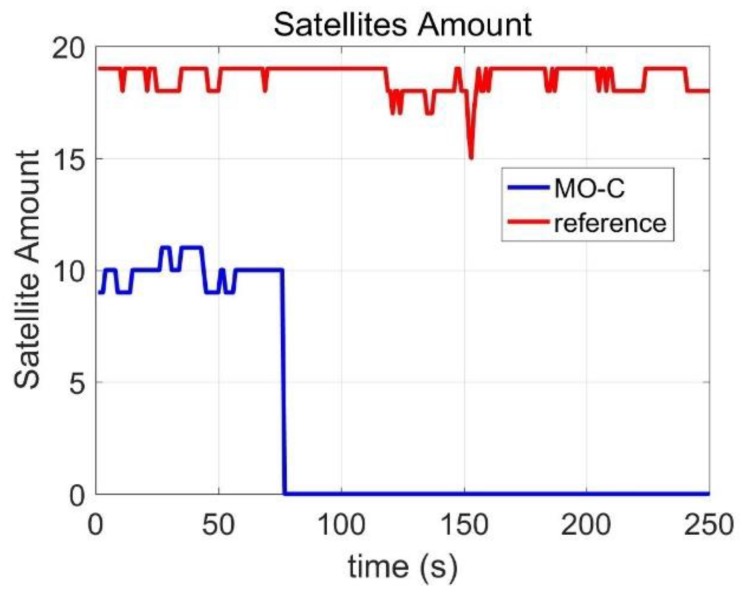
Satellites amount.

In this part, we integrated into the BDS/MINS coupled navigation system for improving its positioning accuracy during a signal outage. Figure 15 presented the field-testing trajectory in Google maps. The BDS satellite amount of change was presented in Figure 16. The red line represented the in-view satellite amount of GPS and BDS, which was employed as the reference. The blue line represented the BDS satellite amount employed in the experiment. At 75 s, the antenna was removed to simulate the signal outage. Therefore, the satellite amount was zero after 75 s. 

**Figure 17:**
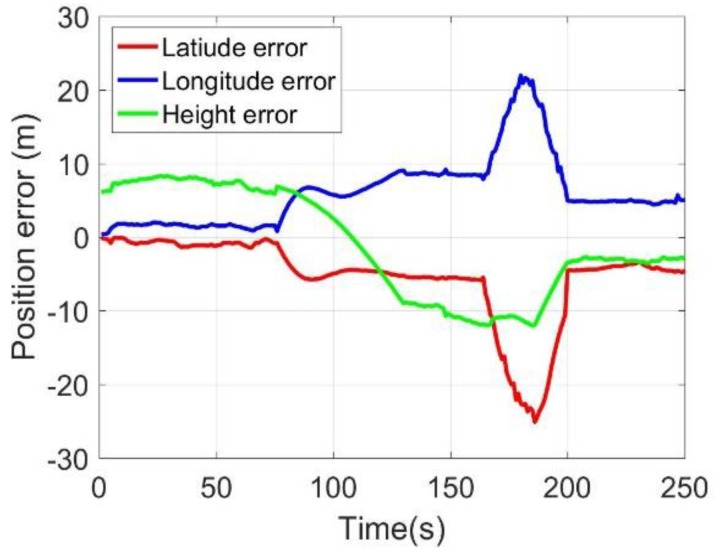
Position errors.

**Figure 18:**
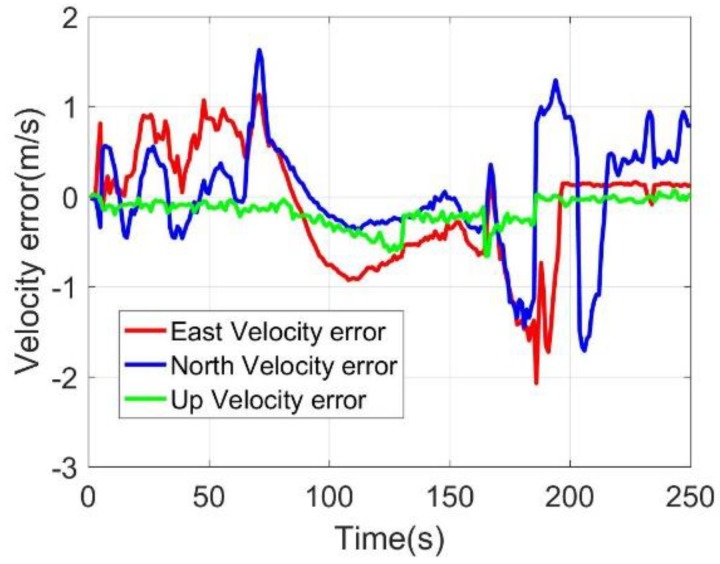
Velocity errors.

Position and velocity errors were presented in Figure 17 and Figure 18. During 0–75 s, the system worked on BDS/MINS integration mode, and the position and velocity errors were within the normal range. The system worked on GNSS/MIMU/Odometer mode during 0–75 s, the benefits could be summarized as follows: firstly, the GNSS provided the initial position and velocity information for the MIMU, and the GNSS velocity was helpful for the attitude estimation; secondly, the odometer could also help the navigation solutions estimation, and in this mode, some parameters of the odometer could be estimated from the reliable navigation solutions. 

After the 75 s, the BDS antenna was removed to simulate the signal outage and to assess the performance of the MC-O. During 75 s and 165 s, the position errors experienced a minor decrease; however, the position errors still kept within 10 m. After 165 s, the odometer was disconnected for the assessment of the M-C performance. The position and velocity errors obtained a dramatic decrease. The latitude and longitude errors were over 20 m. However, the height errors still kept within 10 m, which demonstrated the effectiveness of the up velocity constraint. After 180 s, the odometer was re-connected to the system, and the positioning and velocity errors converged quickly to normal range.

## 4. Discussion

Our experimental results demonstrated that the odometer and the state constraints were effective for suppressing the positioning errors of the MIMU while GNSS was unavailable. The odometer was effective for reducing the errors of the vehicle moving direction, and the constraints also performed well in reducing the height errors. During the 90 s testing time, the MO-C three-dimensional position errors could keep within five meters above IMU. However, we thought the following work was worthy of further investigation:

(1) In the above experiments, the testing time was 90 s, and the position errors would diverge due to the odometer errors and the heading angle errors. In the MO-C, there were no constraints for the heading angle. Other sensors or methods, providing better heading angles, could certainly improve the MO-C position accuracy while GNSS was unavailable for a long time.
(2) In the experiments, we removed the GNSS antenna for simulating the signal outage for assessing the MO-C, in fact, in urban areas, although part of the GNSS satellites were blocked by the surrounding buildings, there were still a few satellites in view. However, there were not enough for generating precise three-dimensional navigation solutions, the remaining satellites might be helpful in the MO-C for aiding the navigation solutions estimation.
(3) Although the NHC and odometer were effective during the GNSS signals outage, it was still necessary for GNSS/MIMU/odometer integration system, while the GNSS was normal, some MIMU and odometer parameters could be estimated and calibrated, which could help reduce the positioning errors during GNSS signal outage.

## 5. Conclusions

In this paper, we present a comprehensive investigation of the MIMU/odometer integrated navigation system with vehicle state constraints. The algorithm is described and listed in detail. Abundant experiments were conducted for evaluating and comparing the performance of the MO, M-C, and MO-C methods. We could conclude that:

(1) Odometer was effective for reducing the latitude and longitude errors, however, it has almost no influence on height accuracy.
(2) These constraints were effective for the height error reduction, but its influence on the latitude and longitude errors were related to the moving direction of the vehicle.
(3) With the odometer and constraints aiding, the heading angle heavily affects the accuracy of the navigation solutions. If the heading angle could be determined precisely, the multi-sensor fusion method could provide long-time three-dimensional navigation solutions without GNSS.
(4) This paper firstly presented the implementation and evaluation of these methods in the BDS/MIMU loose integration system, and the satisfying results could support the BDS for vehicles in urban areas.
(5) The methods discussed in the paper could also be implemented in a BDS chip receiver, and MIMU could be connected to the BDS chip-scale receiver for improving the reliability and robustness of the navigation solutions.

## Figures and Tables

**Table 1:** Inertial Measurement Unit (IMU) specifications.

**Gyroscope**	Bias stability (degree/h)	≤3 degree/h
	Scale factor nonlinearity (ppm)	≤200 ppm
	White noise (degree/h)	0.1 degree/h
**Accelerometer**	Bias stability (mg)	0.1 mg
	Scale factor nonlinearity (ppm)	≤150 ppm
	White noise (mg)	0.05 mg

**Table 2:** Positioning and velocity error comparison.

		Latitude (m)	Longitude (m)	Height (m)	East Velocity (m/s)	North Velocity (m/s)	Up Velocity (m/s)
MIMU	90s error	480.1	−150.6	−95.37	−1.189	7.144	−2.544
	RMSE	234.56	81.31	40.36	0.715	6.121	1.365
MO	90s error	−5.55	−7.30	18.06	−0.152	−0.225	0.931
	RMSE	8.59	7.01	10.34	0.330	0.286	0.416
MO-C	90s error	−5.28	−6.74	3.90	−0.152	−0.223	0.020
	RMSE	8.54	6.93	2.08	0.304	0.286	0.101

**Table 3:** Position and velocity error comparison.

		Latitude (m)	Longitude (m)	Height (m)	East Velocity (m/s)	North Velocity (m/s)	Up Velocity (m/s)
M-C	90s	−25.85	−28.80	−3.06	−0.91	0.27	−0.38
	RMSE	16.662	18.761	4.931	0.686	0.567	0.367
MO	90s	1.74	4.73	−13.35	0.03	−0.06	−0.85
	RMSE	0.876	2.910	7.301	0.162	0.060	0.503
MO-C	90s	1.72	−1.36	−4.38	0.02	−0.04	−0.23
	RMSE	1.911	2.112	5.487	0.142	0.077	0.168
